# hGSuite HyperBrowser: A web-based toolkit for hierarchical metadata-informed analysis of genomic tracks

**DOI:** 10.1371/journal.pone.0286330

**Published:** 2023-07-19

**Authors:** Sumana Kalyanasundaram, Yohan Lefol, Sveinung Gundersen, Torbjørn Rognes, Lene Alsøe, Hilde Loge Nilsen, Eivind Hovig, Geir Kjetil Sandve, Diana Domanska

**Affiliations:** 1 Center for Bioinformatics, Department of Informatics, University of Oslo, Oslo, Norway; 2 Institute of Clinical Medicine, University of Oslo, Oslo, Norway; 3 Department of Microbiology, University Hospital, Oslo, Norway; 4 Department of Tumor Biology, Institute for Cancer Research, Oslo University Hospital, Oslo, Norway; 5 Department of Pathology, University Hospital, Oslo, Norway; 6 Biomedical Informatics, Department of Informatics, University of Oslo, Oslo, Norway; CNR, ITALY

## Abstract

Many high-throughput sequencing datasets can be represented as objects with coordinates along a reference genome. Currently, biological investigations often involve a large number of such datasets, for example representing different cell types or epigenetic factors. Drawing overall conclusions from a large collection of results for individual datasets may be challenging and time-consuming. Meaningful interpretation often requires the results to be aggregated according to metadata that represents biological characteristics of interest. In this light, we here propose the hierarchical Genomic Suite HyperBrowser (hGSuite), an open-source extension to the GSuite HyperBrowser platform, which aims to provide a means for extracting key results from an aggregated collection of high-throughput DNA sequencing data. The hGSuite utilizes a metadata-informed data cube to calculate various statistics across the multiple dimensions of the datasets. With this work, we show that the hGSuite and its associated data cube methodology offers a quick and accessible way for exploratory analysis of large genomic datasets. The web-based toolkit named hGsuite Hyperbrowser is available at https://hyperbrowser.uio.no/hgsuite under a GPLv3 license.

## Introduction

High-throughput sequencing data mapped against a specific build version of a reference genome can be represented as objects with coordinates (referred to as genomic tracks). As this technology grows in availability, large repositories of data are being generated, some private and other public, such as the Hartwig medical foundation database [1], ENCODE [2], IHEC [3], and TCGA [4]. These datasets often contain associated metadata, e.g. cell types, experiment type, replicates etc. Metadata is fundamental in establishing and answering biological questions about the data. Such increasingly large datasets point towards a need to utilize common guidelines to promote sharing and reuse of data, as defined by the FAIR principles [5].

When analysing large collections of tracks based on metadata, the challenge is to be able to compress those results into meaningful ones, making it easy to interpret and draw useful conclusions. Several frameworks and tools have been proposed for performing statistical analysis of collections of genomic tracks, such as FORGE [6], GREGOR [7], LOLA [8], and the GSuite HyperBrowser [9]. However, these existing tools do not consider metadata, and most produce a list of results connected to each input track that has to be processed further to draw conclusions.

To address this issue, we propose a web-based toolkit named hierarchical Genomic Suite HyperBrowser (hGSuite), which enables metadata-informed analysis and interpretation of large collections of genomic tracks. To achieve this, we lean on the concept of a data cube (multidimensional array of values), which is extensively used in other fields, such as business analytics [10]. A data cube enables us to aggregate large amounts of information based on the dimensions found within the metadata of each track (age, sex, treatment, etc.). A given dimension can also be structured hierarchically. For instance, cell types can be structured according to their lineage.

The hGSuite contains a set of open-source, web-based tools to help define hierarchical structures of collections of genomic tracks based on metadata, represented in the hGSuite file format. This file format is designed to handle and share metadata in line with the FAIR principles, following its predecessor, the GSuite format [9, 11]. The tools within the hGSuite enable researchers to perform exploratory statistical analyses of the hGSuite files and represent complex results as a multidimensional cube.

## Materials and methods

### hGSuite HyperBrowser description

The hGSuite HyperBrowser web platform is a set of independent tools that can be used together to cover a full analysis scenario, from data preparation to exploration of results. The pipeline is divided into four major steps that can be used to analyse a collection of genomic tracks based on metadata of interest.

### Data collection

Data can be uploaded or imported from external repositories using the tools provided by GSuite HyperBrowser [9]. If needed, additional metadata can be added to these GSuite tracks. The GSuite files can then be converted to hGSuites using the available metadata. One of many alternatives is to organize data on a local computer into a zip/tar file and directly create a hGSuite from this file.

### Data preparation

Within the web platform, collections of datasets are represented in the GSuite format [9]. The platform includes tools for preprocessing the GSuite tracks into a binary, indexed format in preparation for statistical analyses.

### Statistical analysis and visualization

Analyses are provided for a collection of genomic tracks, as well as for pairwise combinations. Analyses are restricted by the defined hierarchical structure associated with the metadata of the genomic tracks. (Table 1).

**Table 1:** Tools for descriptive statistical and visual analyses of hierarchical genomic tracks.

Category	Tool name	Description	Genomic example
Customize a hGSuite	Modify metadata in hGSuite	Modify specific metadata column of hGSuite	New information about a track based on the patient can be added.
	Filter hGSuite according to metadata	Divides hGSuite based on the information in the metadata column	Group together certain tracks based on metadata and observe the average mutational profile.
	Filter hGSuite based on works in the title column of tracks	Creates or modifies hGSuite based on the name of the track	In a time series data name of the track can indicate the date and time of sequencing. If we want to look at the average change in the mutational profile, we can do so by grouping the tracks based on the date in the title column of the track.
	Concatenate tracks in hGSuite based on metadata	Concatenates tracks in hGSuite according to selected phrases in the selected column	The mutation type where C>T is the same as G>A in the reverse strand, so it would be more meaningful to concatenate the tracks for CT and GA into one track.
	Combine metadata for hGSuite	Merges two metadata columns into one	The gene of interest and the organisms variant (wild-type or knockout) should be combined together to understand the effect of the gene based on the environmental susceptibility.
	Add metadata column in hGSuite	Add new metadata column to an existing GSuite	New information about existing data can be added anytime during the analysis.
Statistical analysis of hGSuite	Expand hGSuite with group summary statistics	Provides an overview of groups such as number of tracks, base-pair coverage, and average length of segments in hGSuite	Overview of the data helps in aiding knowledge of how to formulate research hypothesis.
	Compute data cube for hGSuite	Descriptive statistics such as coverage, observe vs expected coverage for hGSuite defined based on the hierarchy. Also provides plots and tables of the results that can be transposed based on the query.	While having tracks from gene knock-out and wild type data, a detailed overview from different dimensions based on the hierarchy can help define the role of the gene at interest.
	Compute data cube for relations between hGSuites	Provides descriptive statistics such as overlap, normalized coverage and Forbe’s coefficient between two hGSuites defined by hierarchical metadata.	Number of mutations in coding vs non-coding regions.
Visual analysis	Plot flanking bases per mutation counts	Plots the composition of flanking bases in different mutation types	Help in constructing mutation categories for further analysis such as mutational signatures.
	Plot frequency of mutation along chromosome	Generates plot summarising the number of mutations along the chromosome	Visualise mutational hotspots in a particular chromosome.
	Generate rainfall plot	Generates rainfall plots summarising mutation number per location in chromosomes	Visualise the mutational profile along the genome
	Create box plot for tabular file or hGSuite	Present the metadata columns from GSuite or results from tabular file as a box chart.	Visualise the average number of mutations in each group.
	Plot hierarchical clustering	hierarchical cluster plot from columns based on hGSuite	Visualise the hierarchy of the data based on metadata information.

Further descriptions are given on the webpages of the tools themselves, along with demo buttons and links to reproducible examples of how each tool can be used.

### Exploration of results

**Fig 1:**
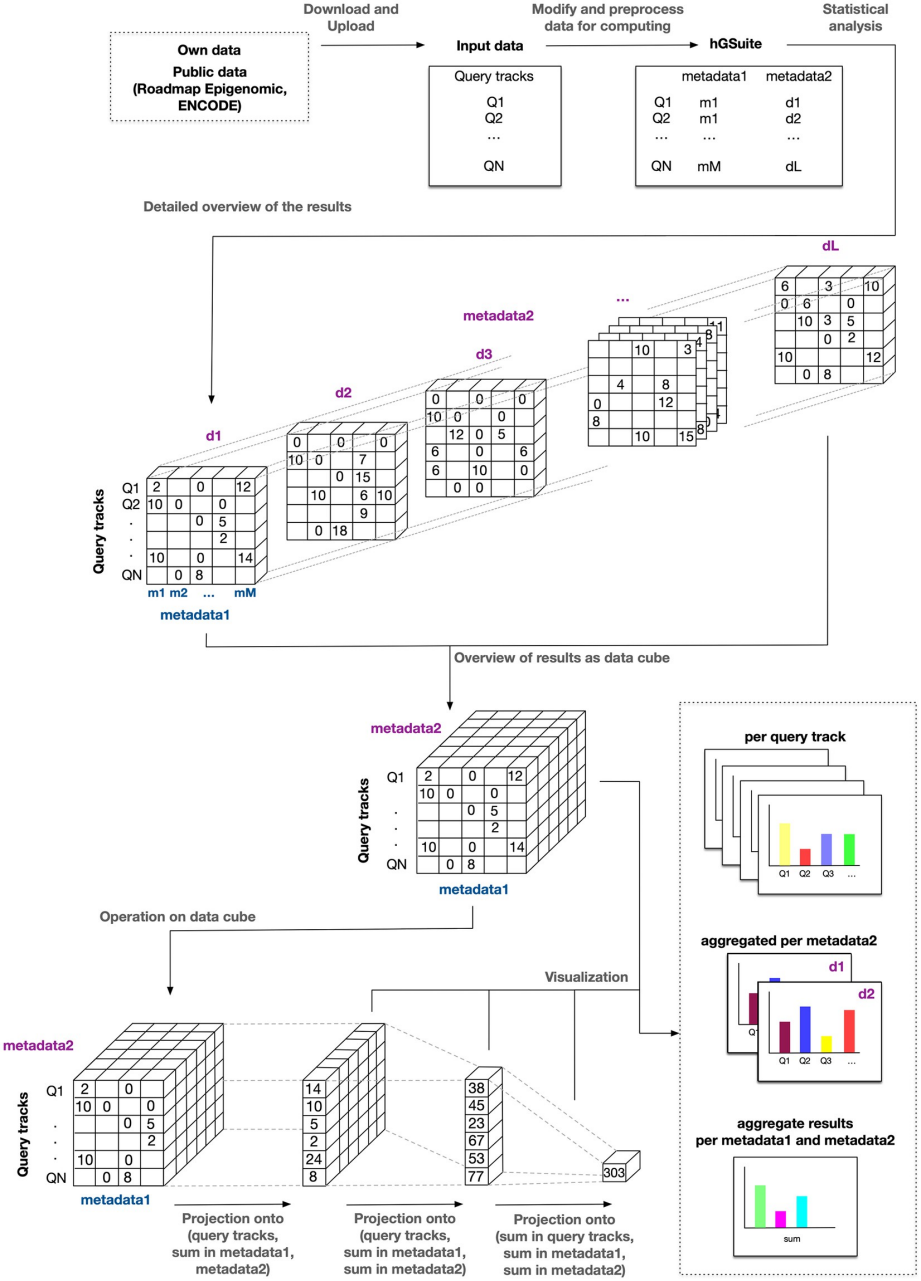
Data cube. Schematic overview of the hGSuite tool that uses the concept of a data cube and its different dimensions. The figure indicates the initial collection and preparation of data, through customization of data using the metadata, to the statistical evaluation for aiding a biological hypothesis. The input is a set of genomic tracks that are converted to GSuite with a hierarchy defined based on the metadata. The data along with metadata can be fitted in a cube that has dimensions based on the hierarchy. The second part of the figure illustrates the different ways in which the data can be viewed in a cube. The cube can then be sliced from different dimensions based on the user query to get the desired output.

Data and analysis results are primarily represented as a data cube Fig 1. This format is suitable for analysing the data from different perspectives, based on the metadata, through operations like slicing, dicing, pivoting, and aggregation [10]. The final results can be viewed as a two-dimensional array, which would enable the user to draw overall conclusions in a time-efficient manner.

## Results

### Case studies

To demonstrate the possible applications for the hGSuite HyperBrowser web platform, two case studies are presented. Data, tools, and a detailed history are provided at https://hyperbrowser.uio.no/hgsuite/, under the Examples section. A visual summary of both studies can also be found in Figs 2 and 3 and S5–S18 Figs.

**Fig 2:**
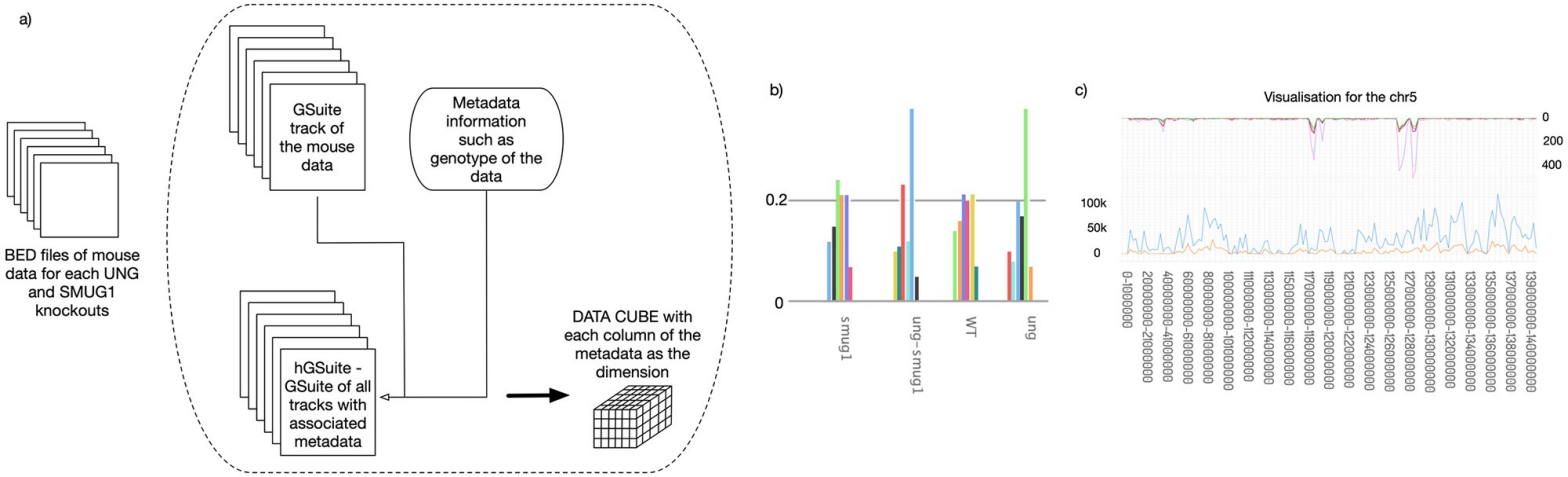
Mouse data. Use case 1 (A-C) exemplify the hGSuite usage. Use case 1 shows the analysis of mutation profiles of different mouse genotypes. A: BED files are converted to GSuite track and metadata information is added to define the hierarchy as a hGSuite. This is then given as input to the tool “Compute data cube for hGSuite” which has the hierarchy of the query track as each dimension. B: Mutation profile of each mutation type in each genotype of the mouse data. C: Rainfall plot of mutational frequency along the genome and mutational frequency plot for one single chromosome.

**Fig 3:**
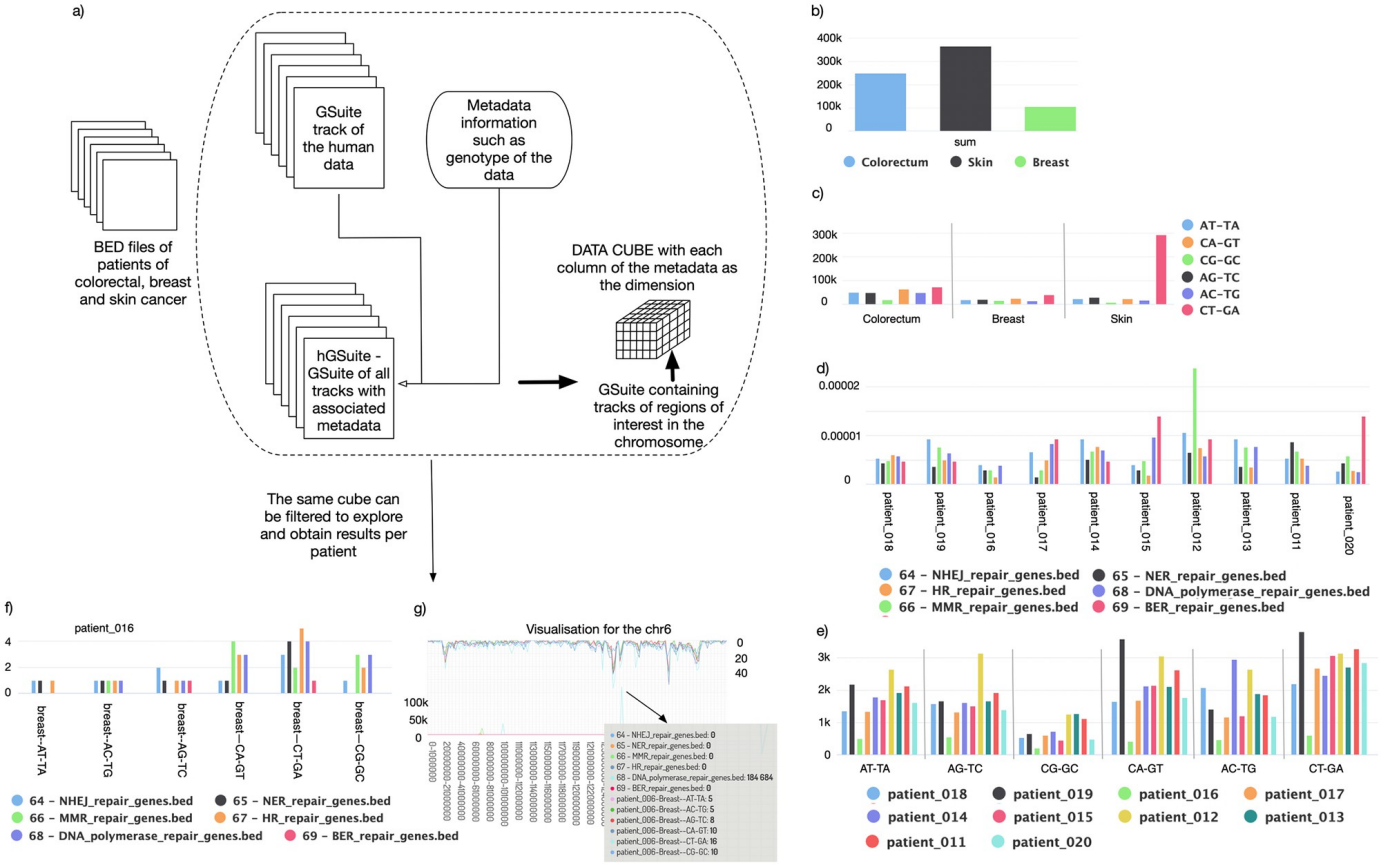
Human data. Use cases 2 (A-G) exemplify the hGSuite usage. Use case 2 shows an analysis of the mutational profile for each patient and the average mutational profile across 3 cancer types—colorectal, breast, and skin cancer. It also shows how the data cube can be filtered to show the frequency of mutation for one patient. A: BED files are converted to GSuite tracks and converted to hGSuite using metadata. The analysis can be done either on just the query track or it can be analysed for overlap with other tracks containing other regions of the genome such as DNA repair genes. B: Average number of mutations across all cancer type. C: Average number of mutations across patients in each cancer type and the average number of mutations of each mutation type across each cancer type. D: Number of mutations for each mutation type across different cancer types in coding regions of reference genome. E: Average number of mutations in DNA repair pathways for every patient with colorectal cancer. F: Average number of mutations of each mutation type for a single patient’s DNA (patient006). G: Frequency of mutations in chromosome 6 for a single patient.

Firstly, we analysed the contribution of DNA repair to antimutagenesis. We have generated three types of murine knockouts (KOs) [12, 14] in the two major U repair enzymes; UNG and SMUG1 [12, 13]. SV40-transformed mouse embryonic fibroblasts (MEF) were serially cultured over 19 passages. MEFs from passage 1 and 15 were subjected to whole genome sequencing. The types of mutations accumulating from passage 1 to 15 were extracted as previously described [14]. BED files from wild type (WT), UNG KO, SMUG1 KO, and UNG-SMUG1 double KO mice were used to look at the mutation profiles with the data cube (Fig 2A–2C). The genotypes of the mice were used as one dimension of the cube. We also split the mouse tracks based on the mutational types, which were used as another dimension of the cube. From S1 Fig, the first observation that can be made is that there was little to no difference between the number and type of mutations accumulating in WT and SMUG1 KO MEFs. We also observed that more mutations accumulated in UNG KO MEFs. As expected, C>T and G>A transitions, most likely originating from spontaneous cytosine deamination, were by far the dominating class of mutation. However, mutations at AT base pairs were also higher in the UNG KO. Uracil misincorporated opposite adenine are read as thymine and are therefore not directly mutagenic. Mutations at AT pairs might therefore suggest that these lesions might be misprocessed. Interestingly, all mutation classes except A>C-T>G were reduced in UNG-SMUG1 double KO. UNG and SMUG1 both contribute to uracil repair [13], but the reduced mutation accumulation in double KO cells challenges the idea that these enzymes are fully compensating for each other. Instead, our data suggest that SMUG1 initiates error-prone processing of uracil residues that are preferentially repaired by UNG. Figure S3 Fig shows the rainfall plot of the genome from which we see a dominance of C>T and G>A mutations (green), which are found in high density within chromosome 13, as well as two different locations within chromosome 5 for the UNG KO. Premutagenic uracil residues are not expected to be introduced homogeneously along chromosomes as the cytosine deamination rates are higher in single-stranded DNA [15]. Similarly, the accessibility of DNA repair enzymes are likely to depend on chromatin compaction. To visualize mutation distribution along chromosomes we developed a tool where mutation frequency (number of mutations per bin) were mapped together with other genomic features, such as exons or CpGs. From figure S4 Fig, we observe that our peaks of interest in chromosome 5 primarily coincide with exons (blue line) and not CpG islands (orange).

For the second biological example, we obtained BED files of somatic nucleotide variants of 30 samples from the Hartwig cancer database [1]. The dataset consisted of 10 patients each for colorectal, breast, and skin cancer. The mutation profile of each patient was studied for preferential occurrence in specific regions of chromosomes, such as coding, non-coding, and regulatory regions, both individually and also based on cancer type. The results are shown in S2 Fig. To compare general mutational profiles, such as the average number of mutations based on cancer type, we aggregate the results across the “patientID” dimension and display the value for each based on the primary tumor type. Fig 3(A)–(3E), S5–S7 Figs show that consistent with large datasets [15], the mutational burden of skin cancer patients is relatively higher compared to breast and colorectal cancer patients. Similarly, when we aggregate the results based on mutation type for each cancer type dimension and the transpose of it (S6 and S7 Figs, respectively), we observe the relative occurrence of different mutation types across cancers. As expected, the C:G to T:A mutation is the most common across all cancer types due to the deamination of cytosine or 5-methylcytosine. For the next analysis, we observed how the frequency of mutations varies along chromosomes, and correlates with functional features such as coding and non-coding regions. To do this, we compare the GSuite of the patients against a GSuite containing information about coding and non-coding regions of the reference (hg19) genome. The results for the coding region mutational profile for different mutation types are shown in S8–S10 Figs. S8 Fig shows the result of the aggregated average overlap in the coding region for each mutation type across cancers. From S10 Fig, we observe that skin cancer patients have the highest average of C>T-G>A mutations in the genome and in the coding regions. Ultraviolet light is the major driver of mutagenesis in cutaneous melanomas, which produces a distinct mutational signature with C>T and CC>TT dinucleotide transitions [15] which explains the high occurrence of the C>T-G>A mutation type. Similarly, in colon cancer patients, after the C>T-G>A mutation, it is C>A-G>T that appears most frequently, a common cause of driver mutations in e.g. KRAS, in colorectal cancer patients [16]. As a next step in understanding our dataset, we wanted to study the co-occurrence of mutations in the chromatin state of a given cell type, since mutations in open chromatin regions are a prognostic factor in various cancers [17]. Hence, understanding the effects of individual mutations in the chromatin state of a given cell type may allow the identification of novel cancer-associated regulatory genes or sequences [17]. GSuites of DNAseq and ATACseq for cell lines associated with each cancer type were obtained from ENCODE and used in the hGSuite. The compute relations between two hGSuite tools were used to obtain an average occurrence of mutations in the open chromatin regions (S11 and S12 Figs). This provides a broad view of co-occurrence in each cell line for mutations of a patient with a particular type of cancer. A failure in DNA repair pathways and cell cycle checkpoint networks that cooperate to ensure the maintenance of genetic stability can lead to tumor growth [18]. Hence, the next set of questions we want to ask about our data is based on DNA repair genes and their associated pathways. To do so, we downloaded the location of DNA repair genes. The genes are classified based on the DNA repair pathways such as mismatch repair (MMR), base-excision repair (BER), non-homologous end-joining repair (NHEJ), nucleotide excision repair (NER), homologous recombination (HR), and the set of DNA polymerase genes. The GSuite of patients was compared with the GSuite for DNA repair pathway genes consisting of all 6 different pathways. The result for colorectal cancer is shown in S14 Fig, where we get the average normalized count for each mutation type across patients with colorectal cancer. When we aggregate along the first GSuite of patients and select colorectal cancer as the primary tumor location, we notice that MMR and NHEJ are the two repair pathways that show a higher number of mutations. It has been shown that approximately 15–18% of colorectal carcinoma show mismatch repair deficiency [19–22] and up to 3% of tumors develop in association with Lynch syndrome that also results from mutations in MMR genes, most commonly MLH1, MSH2, MSH6 or PMS2 [23]. DNA double-stranded breaks (DSBs) are genotoxic carcinogens, and the NHEJ allows DNA recovery by direct joining [24]. It has been shown that increased mutations within NHEJ genes increase the risk of colorectal cancer [23]. Among breast cancer patients (S15 Fig), we observed a high frequency of mutations in the base-excision repair pathway (BER). While observing skin cancer (S16 Fig), it clearly shows an increase in mutations along all the pathways compared to the other two types of cancer which fits well with the previous observations that show the highest mutation burden.

The above case study can also be done for each patient (illustrated in Fig 3(D), 3(F) and 3(G)) and can be useful in developing treatment strategies. We have done this with a breast cancer patient having an odd mutation profile and found that a highly mutated region within this patient mapped to the DNA polymerase genes. S17 and S18 Figs shows the rainfall plot and frequency plot profile for patient 6 for each chromosome. Overall, these results show the hGSuite’s ability to aid in both basic and personalized research.

### Comparing hGSuite to other programming tools

The main approach in the field of bioinformatics has been to download data from multiple databases and restructure the data to define the hierarchy. Downloading and re-integrating the tracks or collection of tracks as needed quickly becomes time-consuming. To illustrate the benefits of using the hGSuite, we provide an example of an analysis to get an overall average mutation profile of the tracks based on any dimension such as cancer type, mutation type, or per patient. The results of the analysis of overlap between certain genomic regions of the chromosome and the tracks from any of the above-mentioned dimensions are shown and explained. The screencast (https://bit.ly/hGSuite) illustrates the analysis using the hGSuite while the R code shows the equivalent analysis. We compare it to the equivalent R analysis (S1 File).

The different statistical and visualization tools in the hGSuite enable performing analysis on multiple tracks by simple repetition of computations on each track. The results of the analysis are saved in the data cube making it flexible for the user to analyse and visualize the results in any dimension defined using the metadata. In the hGSuite this takes 5 clicks as shown in the screencast. In R, the data require a manual download, and the code is written for every dimension separately. The code presented does not necessarily represent the optimal way of solving it with R, but we do believe it to be representative of how an analyst might typically approach such an analysis. Notably, it would take more than 100 lines of code to achieve the same result we would get in 4 clicks with hGSuite.

## Discussion

The hierarchical Genomic HyperBrowser is a comprehensive system for descriptive statistical analysis of genomic tracks along with associated metadata. This tool when combined with several other tools within the HyperBrowser and other tools outside of the HyperBrowser has a powerful way of analysing and representing the genomic data. It gives an overall impression and idea about the data through statistical and visual means, thus aiding the formulation of relevant hypotheses. The hGSuite methodology is built based on the concept of a data cube and is currently focused on track analysis, however, the methodology can be adopted for other types of analysis. The GSuite HyperBrowser platform provides a comprehensive solution for integrating and analyzing track collections throughout the genome, while hGSuite helps in analysing the tracks based on metadata-based groupings. The hGSuite platform is based on the Galaxy platform [25], making it more accessible for integrating with workflows for other types of data such as metabolomics or proteomics data, which can subsequently be used as metadata information for hGSuites. Programming in languages like python and R can help us obtain the same results as hGSuite, but requires prior knowledge of programming, and may require substantially more effort. S1 File provides R code for doing the first few steps of the same analysis as in case study 2. Several lines of R code is needed to do the same thing that can be done in a few clicks in the hGSuite (S1 Video). This shows that the hGSuite may be useful to programmers and non-programmers alike, ensuring reproducibility more time-efficiently than R. The data used to illustrate the ability of the hGSuite does not necessarily explore its potential to the full extent. In case study 2 with the human cancer genome, we could have used more metadata like age, gender, and tumor purity to build a much deeper hierarchical system. Also, any type of genomic data that has a hierarchy can be exploited for further analysis or to formulate hypotheses using the hGSuite. The hGSuite also makes it easy to add more data or metadata information to the existing GSuite throughout the analysis. Furthermore, the hGSuite acts as a one-stop shop for a range of genomic investigations that can be addressed through a combination of genomic tracks and their associated metadata.

## Conclusion

In this paper, we have presented hGSuite HyperBrowser and its associated data cube methodology. We have then illustrated its diverse applications for investigating various dimensions of whole-genome or whole-exome data. The demonstration illustrates hGSuite’s ability to perform analyses on an exploratory level, as well as for deeper analyses. With the functionalities provided, we believe hGSuite, paired with the GSuite HyperBrowser platform, will enable greater access and versatility to explorative large-scale genomic analyses. To our knowledge, hGSuite HyperBrowser is the only option for performing all of the aforementioned analyses within a single platform without any need for time-consuming manual transformation of metadata.

## Supporting information

S1 Fig Mutation type frequency. Shown is the frequency of each mutation type across different knockout mouse data.
(TIF)

S2 Fig Rainfall plot of mouse genome. The rainfall plot illustrates the genomic position (x axis) versus the genomic distance (y axis) where the genomic position represents the position along the genome, starting from the beginning of chromosome 1 (left hand-side) to the end of chromosome y (right hand-side). This plot shows the genome for each genotype.
(TIF)

S3 Fig Single rainfall plots. The rainfall plot illustrates the genomic position of the UNG genotype by selecting ‘single’ as the plot type.
(TIF)

S4 Fig Frequency of mutation along a chromosome. Frequency plots for each individual chromosome.
(TIF)

S5 Fig Average number of mutations across cancer types. Shows the average number of mutations for each cancer type.
(TIF)

S6 Fig Average number of each mutation type for each cancer type. Shows the average number of mutations per mutation type across the 3 different cancers.
(TIF)

S7 Fig Average number of each mutation type for each cancer type. Shown is the transposed table of S6 Fig that shows the average number of mutations for each mutation type for each cancer type.
(TIF)

S8 Fig Average mutation in the coding region for each cancer. Shows the number of each mutation type for each patient for each cancer type. The first is colorectal cancer, the second is breast cancer and the third is skin cancer.
(TIF)

S9 Fig Total number of mutations in the coding region for each mutation type for each patient. Shows the transpose of S8 Fig that shows the number of mutations for each mutation type for each patient in exons. It is shown for each cancer type.
(TIF)

S10 Fig Average mutations in the coding region for each mutation type across cancers. The plots show average mutations for each mutation type in coding regions.
(TIF)

S11 Fig Chromatin state of each patient in colorectal cancer. Shows the number of mutations in colorectal cancer cell-line for each patient.
(TIF)

S12 Fig Chromatin state of each patient with skin cancer. Shows the number of mutations in skin cancer cell-line for each patient.
(TIF)

S13 Fig Number of mutation in genes in a DNA repair pathways for each cancer type. (TIF)

S14 Fig Number of mutations in each mutation type across different DNA repair pathways for colorectal cancer patients. (TIF)

S15 Fig Number of mutations in each mutation type across different DNA repair pathways for breast cancer patients. (TIF)

S16 Fig Number of mutations in each mutation type across different DNA repair pathways for skin cancer patients. (TIF)

S17 Fig Distribution of mutation type in the DNA repair pathways. Shows the distribution of each mutation type for patient006 for each of the DNA repair pathways.
(TIF)

S18 Fig Frequency of mutation along chromosome 6 for patient006. Show the frequency of distribution of mutations along chromosome 6 for patient006.
(TIF)

S1 File Rcode. The R code replicates a few steps of the analysis. This is done mainly to compare the ease of using hGSuite tool over other programming languages.
(R) Click here for additional data file.

S1 Video Quick user guide video tutorial. A screencast video demonstration for a quick start for using the hGSuite tool. It illustrates the first few steps of the analysis of the human case study explained in the manuscript.
(DOCX)
