# Supplementary figures and images for: hGSuite HyperBrowser: A web-based toolkit for hierarchical metadata-informed analysis of genomic tracks
Source: PLoS One. 2023 Jul 19;18(7):e0286330. doi: 10.1371/journal.pone.0286330 (PMC10355376; doi:10.1371/journal.pone.0286330)

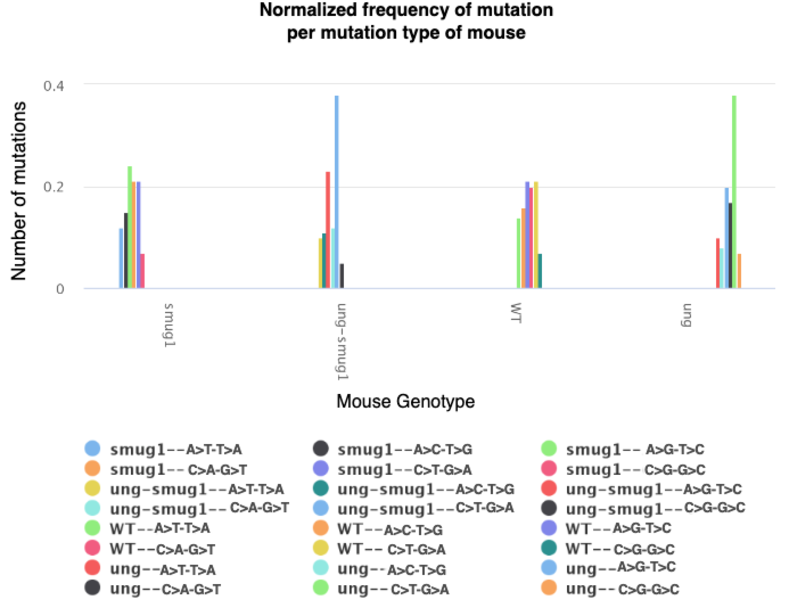

Supplement: S1 Fig — Shown is the frequency of each mutation type across different knockout mouse data. (TIF) [file pone.0286330.s001.tif]

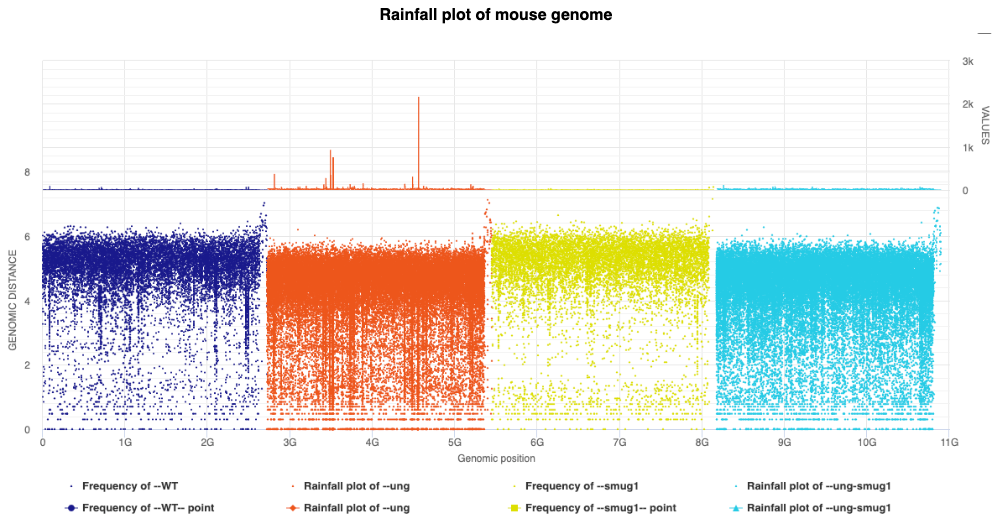

Supplement: S2 Fig — The rainfall plot illustrates the genomic position (x axis) versus the genomic distance (y axis) where the genomic position represents the position along the genome, starting from the beginning of chromosome 1 (left hand-side) to the end of chromosome y (right hand-side). This plot shows the genome for each genotype. (TIF) [file pone.0286330.s002.tif]

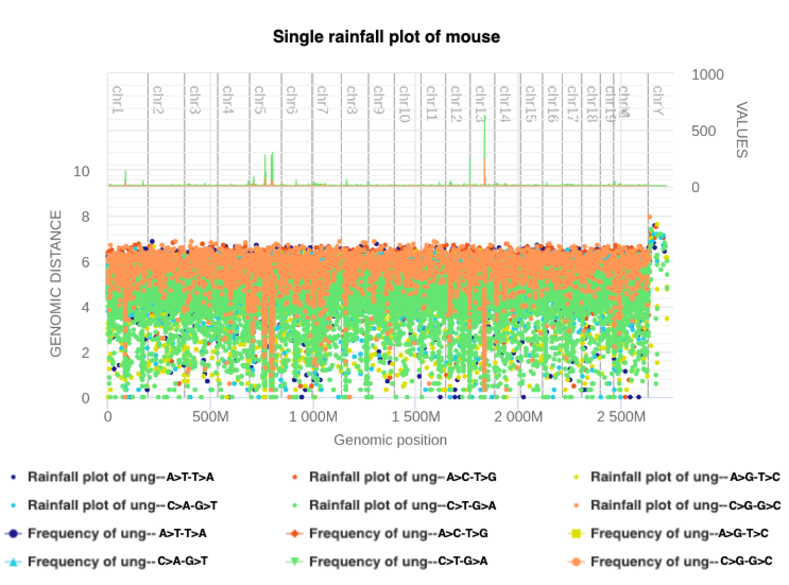

Supplement: S3 Fig — The rainfall plot illustrates the genomic position of the UNG genotype by selecting ‘single’ as the plot type. (TIF) [file pone.0286330.s003.tif]

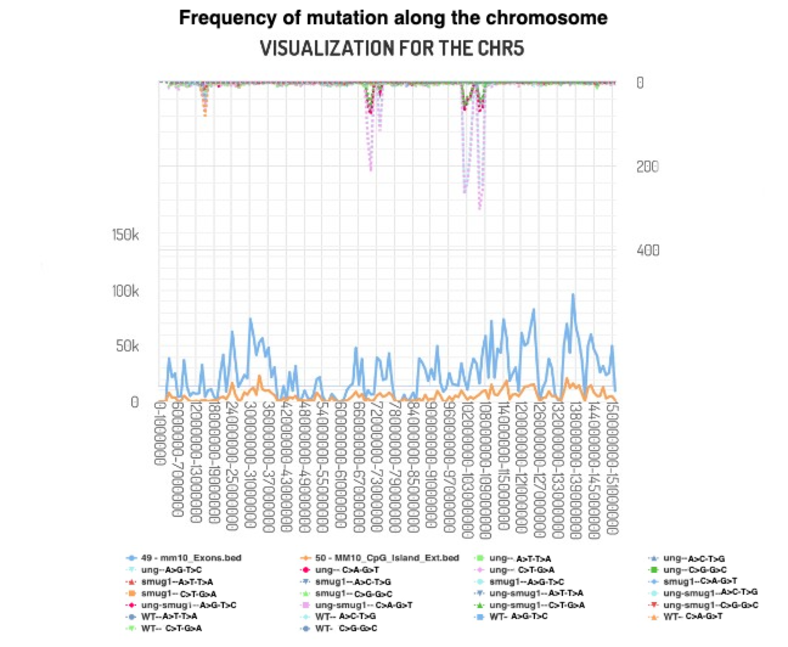

Supplement: S4 Fig — Frequency plots for each individual chromosome. (TIF) [file pone.0286330.s004.tif]

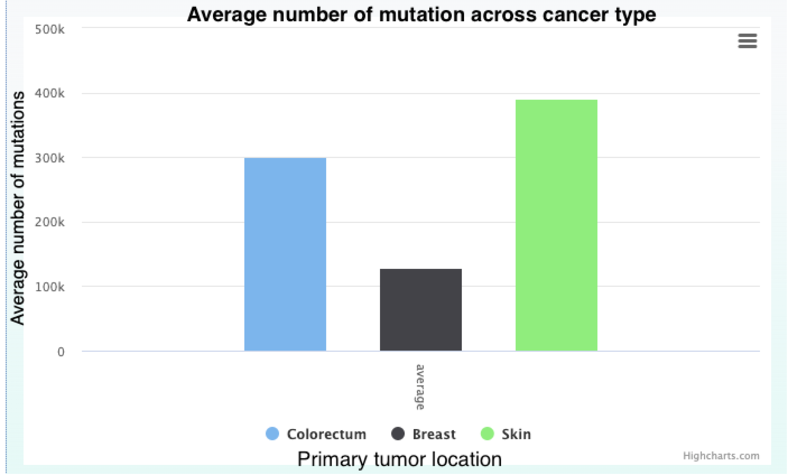

Supplement: S5 Fig — Shows the average number of mutations for each cancer type. (TIF) [file pone.0286330.s005.tif]

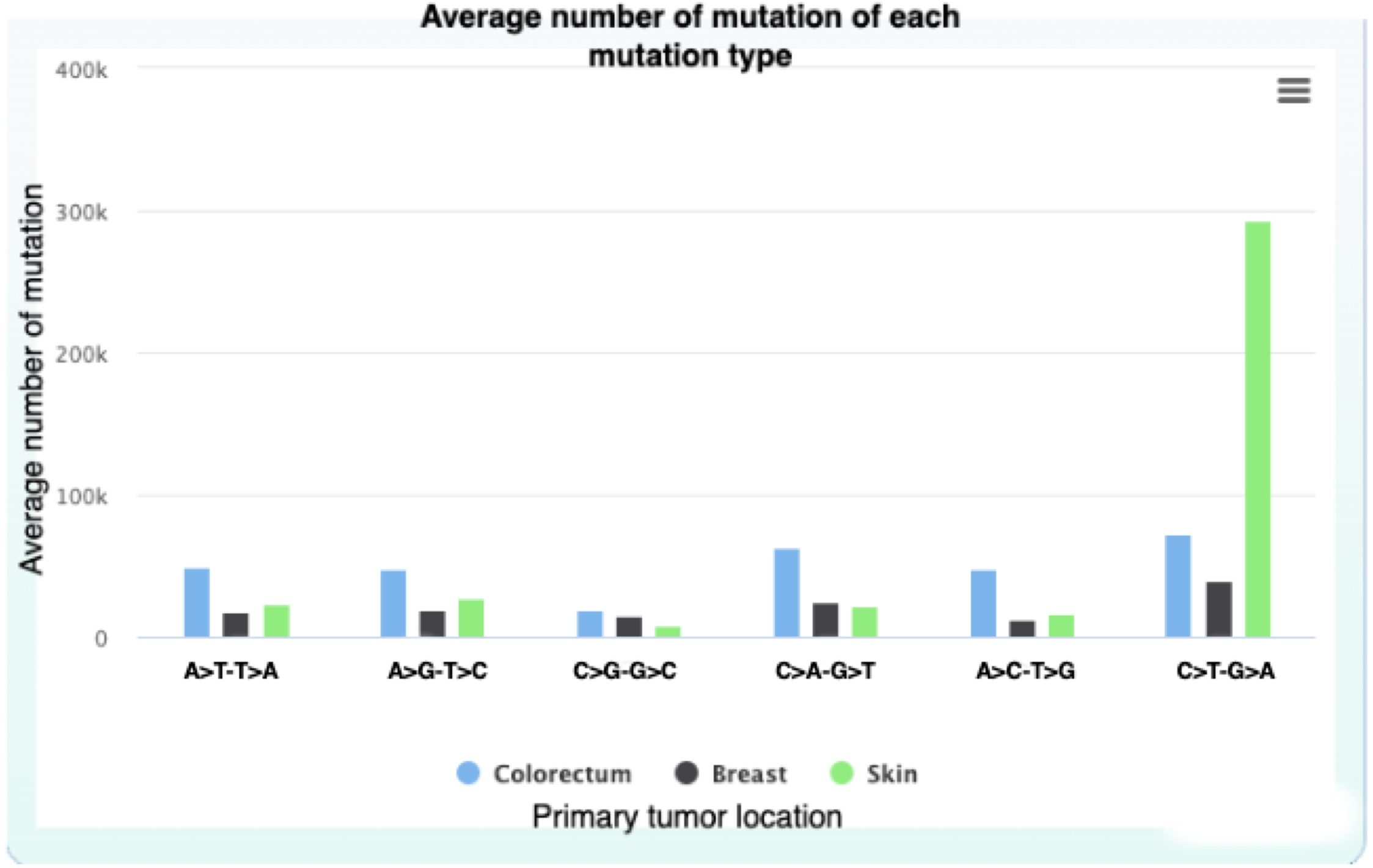

Supplement: S6 Fig — Shows the average number of mutations per mutation type across the 3 different cancers. (TIF) [file pone.0286330.s006.tif]

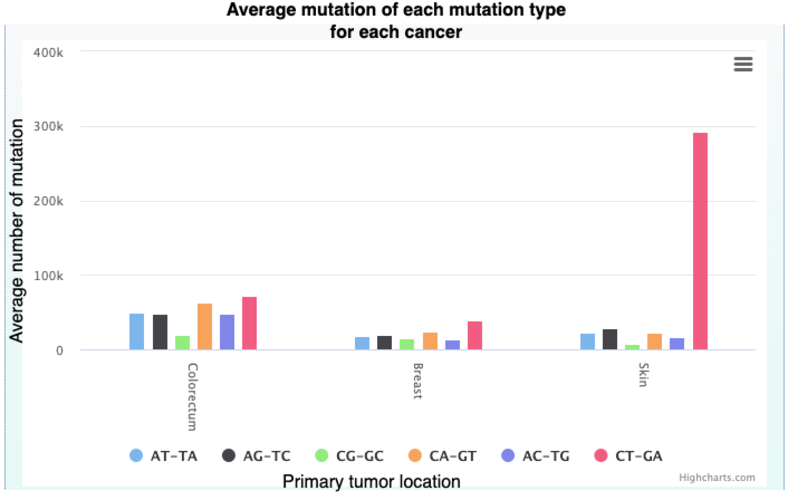

Supplement: S7 Fig — Shown is the transposed table of S6 Fig that shows the average number of mutations for each mutation type for each cancer type. (TIF) [file pone.0286330.s007.tif]

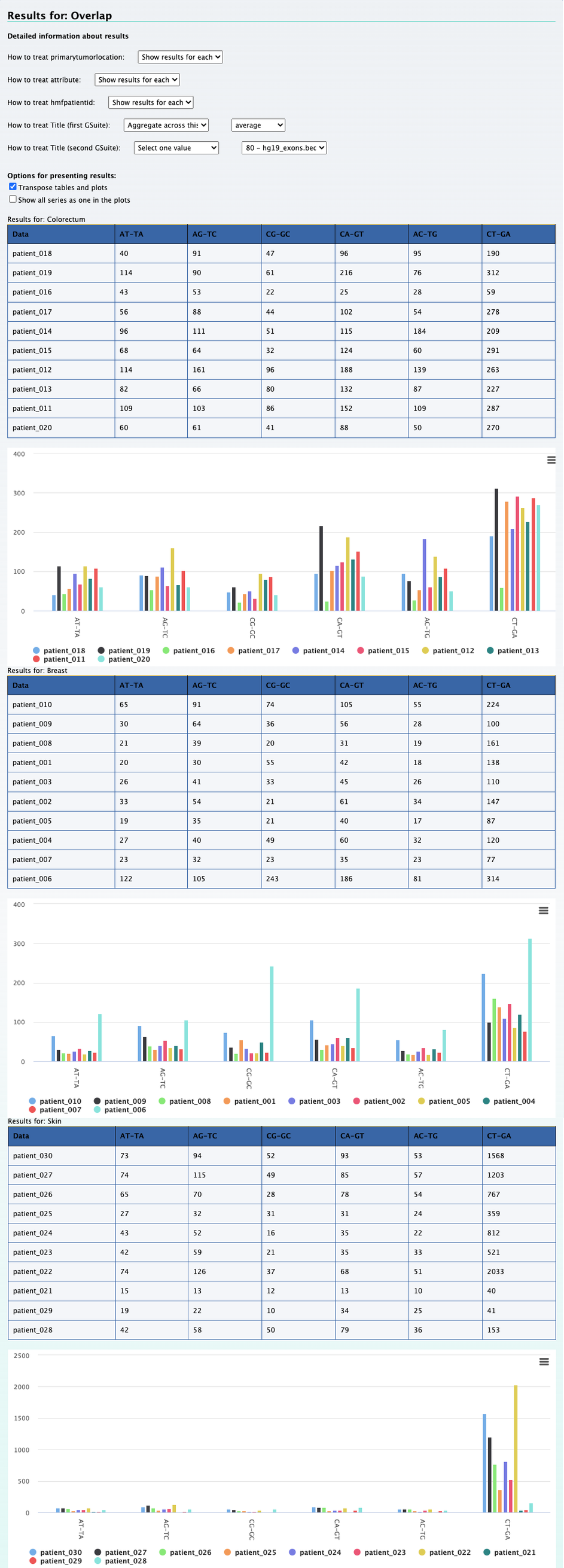

Supplement: S8 Fig — Shows the number of each mutation type for each patient for each cancer type. The first is colorectal cancer, the second is breast cancer and the third is skin cancer. (TIF) [file pone.0286330.s008.tif]

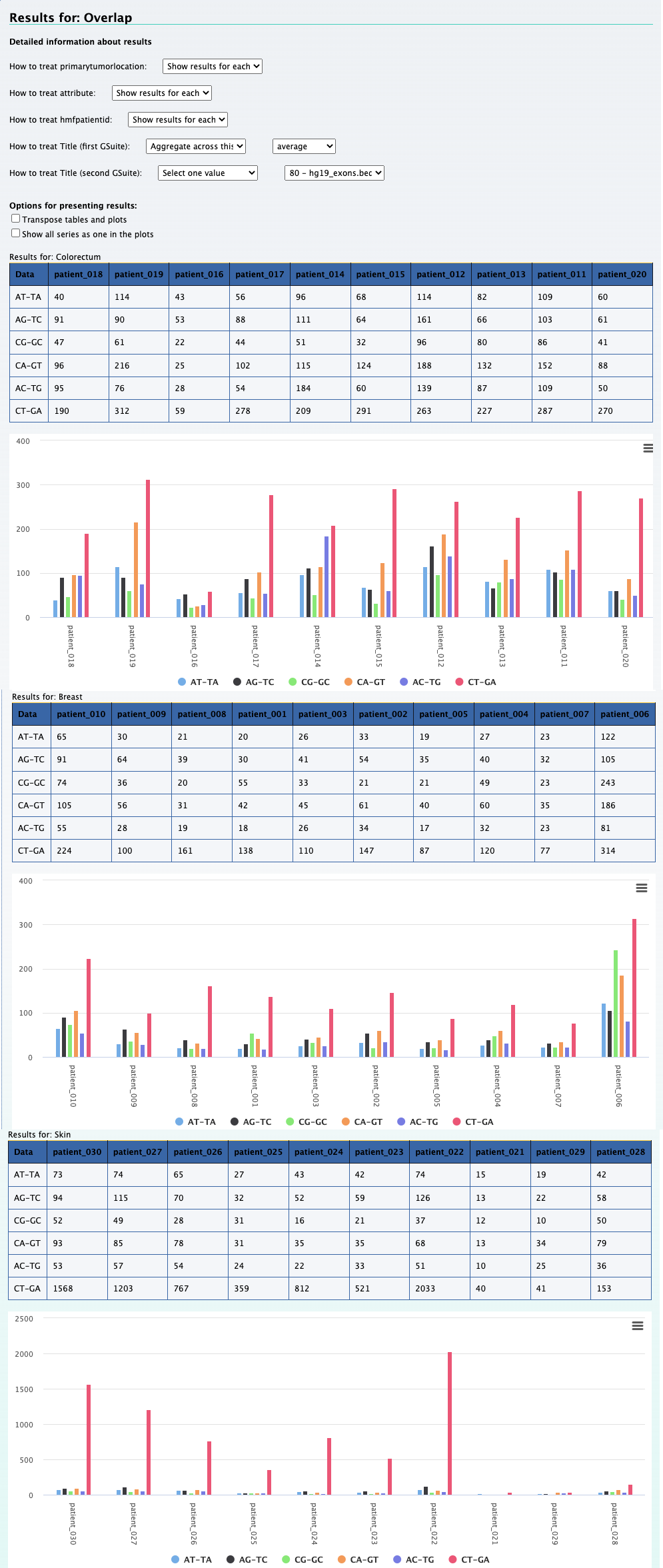

Supplement: S9 Fig — Shows the transpose of S8 Fig that shows the number of mutations for each mutation type for each patient in exons. It is shown for each cancer type. (TIF) [file pone.0286330.s009.tif]

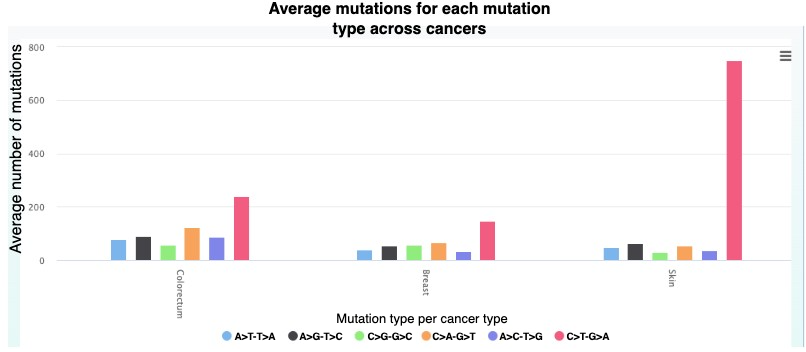

Supplement: S10 Fig — The plots show average mutations for each mutation type in coding regions. (TIF) [file pone.0286330.s010.tif]

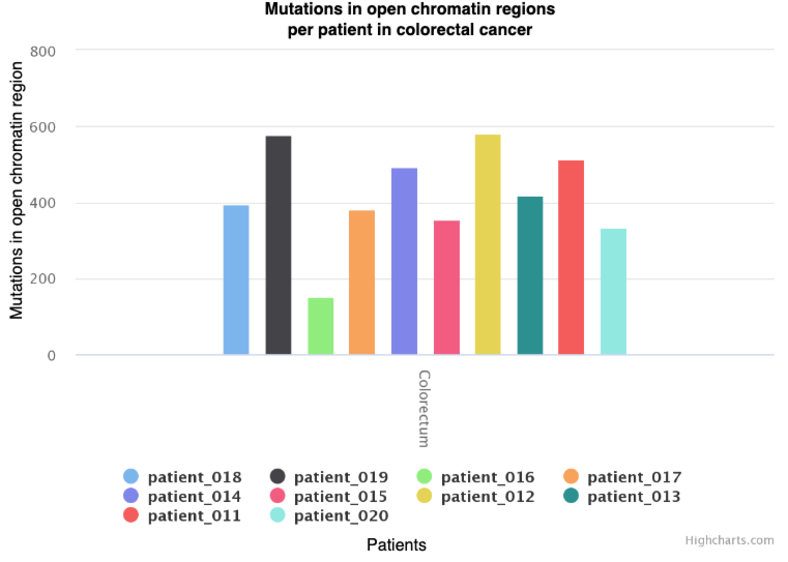

Supplement: S11 Fig — Shows the number of mutations in colorectal cancer cell-line for each patient. (TIF) [file pone.0286330.s011.tif]

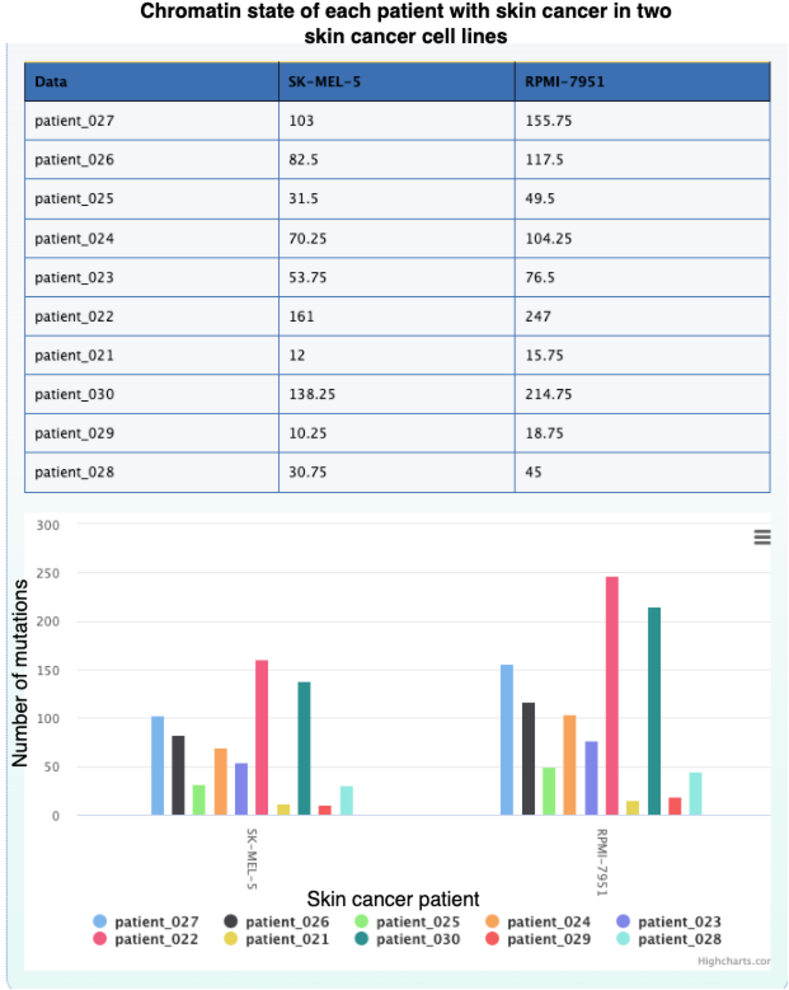

Supplement: S12 Fig — Shows the number of mutations in skin cancer cell-line for each patient. (TIF) [file pone.0286330.s012.tif]

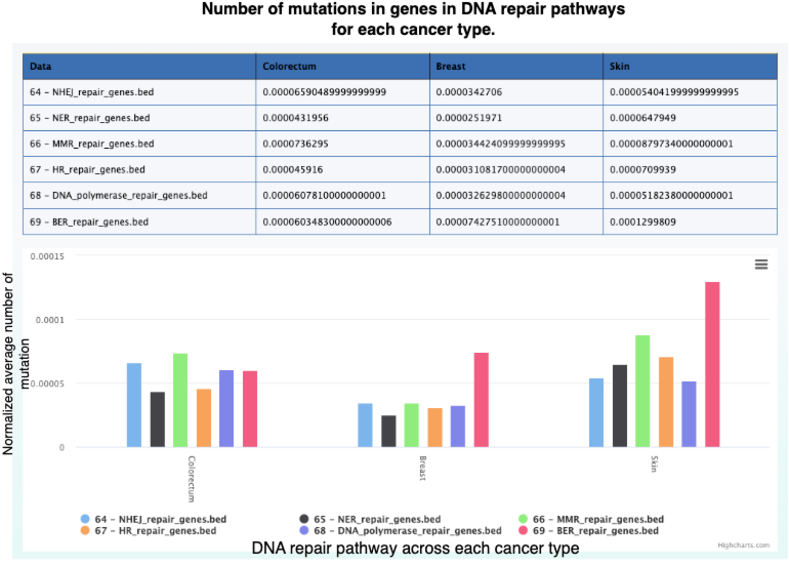

Supplement: S13 Fig — (TIF) [file pone.0286330.s013.tif]

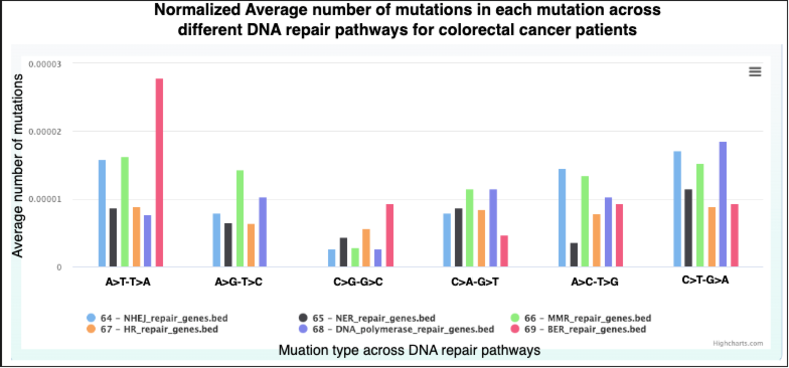

Supplement: S14 Fig — (TIF) [file pone.0286330.s014.tif]

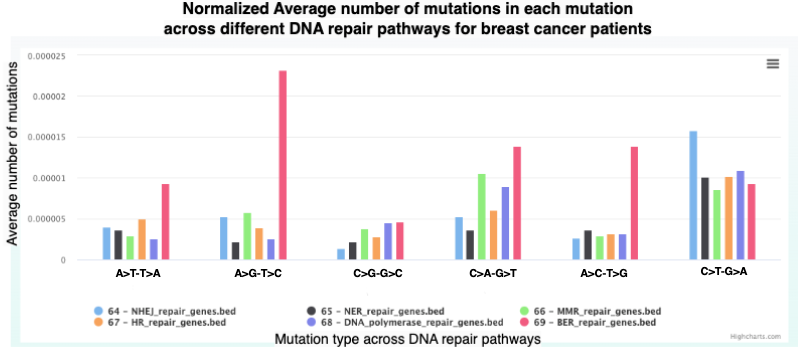

Supplement: S15 Fig — (TIF) [file pone.0286330.s015.tif]

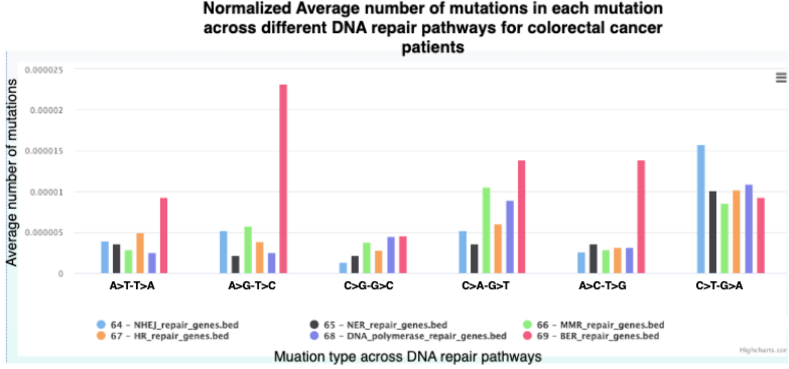

Supplement: S16 Fig — (TIF) [file pone.0286330.s016.tif]

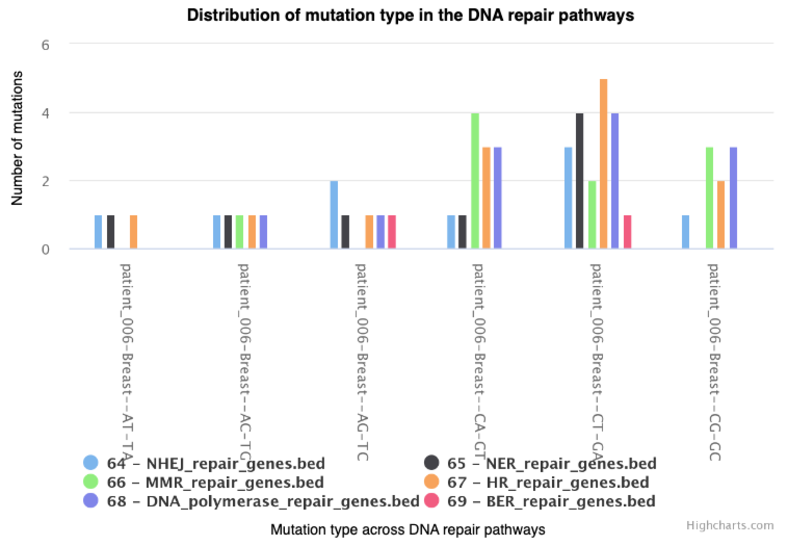

Supplement: S17 Fig — Shows the distribution of each mutation type for patient006 for each of the DNA repair pathways. (TIF) [file pone.0286330.s017.tif]

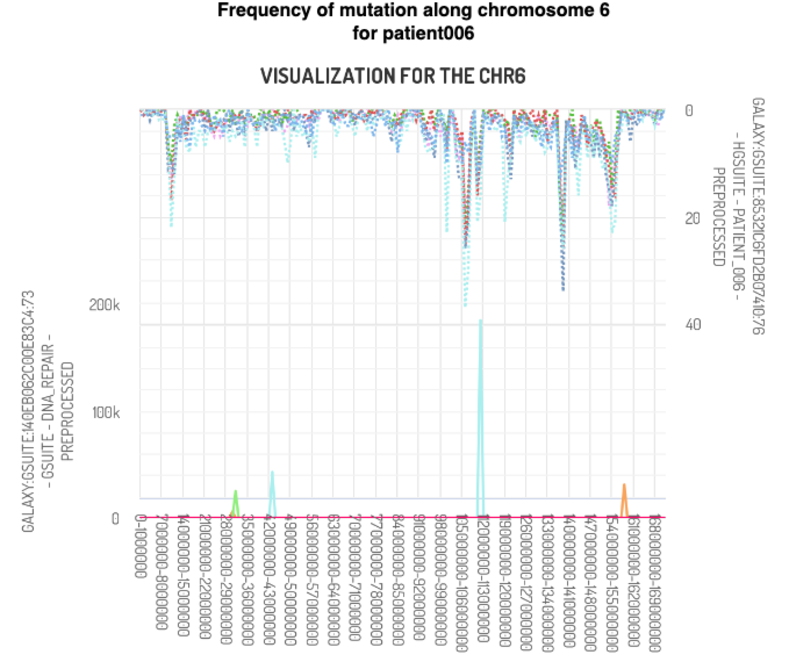

Supplement: S18 Fig — Show the frequency of distribution of mutations along chromosome 6 for patient006. (TIF) [file pone.0286330.s018.tif]
