# Supplementary material for: hGSuite HyperBrowser: A web-based toolkit for hierarchical metadata-informed analysis of genomic tracks
Source: PLoS One. 2023 Jul 19;18(7):e0286330. doi: 10.1371/journal.pone.0286330 (PMC10355376; doi:10.1371/journal.pone.0286330)
Supplement: S1 Video — A screencast video demonstration for a quick start for using the hGSuite tool. It illustrates the first few steps of the analysis of the human case study explained in the manuscript. (DOCX) [file pone.0286330.s020.docx]

Link to the screencast

Short link to the video:

<https://bit.ly/hGSuite>

Full link to the video:

<https://vimeo.com/812434926>
